# An Assessment of Radiological Hazards from Gold Mine Tailings in the Province of Gauteng in South Africa

**DOI:** 10.3390/ijerph13010138

**Published:** 2016-01-18

**Authors:** Caspah Kamunda, Manny Mathuthu, Morgan Madhuku

**Affiliations:** 1 Center for Applied Radiation Science and Tehnology, North West University (Mafikeng), P.Bag X2046, Mmabatho 2735, South Africa; Manny.Mathuthu@nwu.ac.za; 2 iThemba LABS, National Research Foundation, Private Bag X11, WITS 2050, South Africa; madhuku@tlabs.ac.za

**Keywords:** radiological hazard, radionuclide, gold mine tailing, gamma spectroscopy, activity concentration

## Abstract

Radiological hazards associated with exposure to Naturally Occurring Radionuclides Materials from gold mine tailings in the province of Gauteng in South Africa were evaluated. A comparison was made with soil samples from a control area. In this study, gamma spectroscopy was used to measure the activity concentrations of these radionuclides in 56 soil samples from the mine tailings and 10 soil samples from the control area. The average activity concentrations in Bq∙kg^−1^ for Uranium-238, Thorium-232, and Potassium-40 from the mine tailings were found to be 785.3 ± 13.7, 43.9 ± 1.0 and 427.0 ± 13.1, respectively. On the other hand, the average activity concentrations in Bq∙kg^−1^ for Uranium-238, Thorium-232, and Potassium-40 from the control area were found to be 17.0.1 ± 0.4, 22.2 ± 0.5 and 496.8 ± 15.2, respectively. Radiological hazard parameters calculated from these activity concentrations were higher than recommended safe limits. In particular, calculated average values for the external hazard (*H_ex_*) and the internal hazard (*H_in_*) from the mine tailings were found to be 2.4 and 4.5. Both these values were higher than unity, posing a significant health risk to the population in the area.

## 1. Introduction

The environment around us always contains small amounts of Natural Occurring Radioactive Materials (NORMs), which have existed since the formation of the earth. Their availability in the environment is generally at levels that are not potentially harmful to human health. A major concern comes when the levels are elevated as a result of human practices like mining or natural hazards like earth quakes [1].

In nature, mining involves the production of large quantities of waste, which may contaminate soils over a large area, thereby negatively impacting the environment and human health [2]. Mining is one of the major causes of elevation of NORMs concentrations on the earth’s surface causing health risks to humans, especially when inhaled or ingested [3]. The most important NORMs in radiation protection are radionuclides from the Uranium-238 (^238^U) and Thorium-232 (^232^Th) decay series. Potassium-40 (^40^K), a non-series radionuclide, also contributes significantly to human exposure in the environment [4]. Therefore, the knowledge of ^238^U, ^232^Th and ^40^K activity concentrations is important in the evaluation of absorbed doses that can lead to the estimation of their radiological hazard to the population.

The series NORMs produced from these mining activities continue to decay until a stable nuclide is formed. In this decay process, ionizing radiation that produces biological damage to human organs is emitted. For instance, epidemiological studies have shown high mortality rates from respiratory diseases and lung cancer in miners working underground in the Erzmountains of Eastern Europe [5]. More human evidence of the harmful nature of ionizing radiation was also reported by early radiologists and persons working in the radium industry [6]. Both empirical observations and epidemiological studies have consistently shown carcinogenic properties of ionizing radiation. Survivors of the Hiroshima atomic bomb exposed to radiation above one Sievert showed a significant increase in the incidence of leukemia [6].

In South Africa, the gold mining industry has existed for over a century. As a result, mine tailings are littered everywhere, particularly in the Wonderfonteinspruit Catchment Area (WCA), posing a threat to local communities. The communities living around these areas are threatened by radioactive pollution mainly caused by uranium [7]. In 2009, the Gauteng Department of Agriculture and Rural Development reported that the gold-bearing ore in the WCA contained almost ten times the amount of uranium than the gold itself [8]. Although, this was reported on a wide area, there is limited scientific information in the study area about the radiological hazards of NORMs to the population. As a result, a radiological hazard assessment was carried out in order to evaluate the health risk to the population in the mining area.

## 2. Materials and Methods

### 2.1. The Study Area 

The study area is a gold mining area situated about 70 km west of Johannesburg in the province of Gauteng in South Africa. It lies between 26°18’S–26°26’S latitude and 27°23’E–27°31’E longitude. Gold exploration in the area dates back to 1898, and mining has occurred since 1945 [7]. Geologically, the area, approximately 86 km^2^, is located in the West Wits line (Far West Rand) Goldfield of the lower central part of the Wonderfonteinspruit Catchment Area (WCA). Mining activities are engaged in both deep-level (500–4000 m), high-grade underground mining as well as low-grade, surface rock dump mining. The Far West Rand is densely populated because of the presence of these gold mines, and soil that has agricultural value. This WCA forms part of the Witwatersrand Basin, which is the world largest gold and uranium mining basin that covers an area of 1600 km^2^ [7]. The basin has led to a legacy of some 400 km^2^ of mine tailings dams (270 tailings dams and 380 mine residue dumps) containing 6 billion tonnes of pyrite tailings and 600,000 tonnes of low-grade uranium [9]. 

The gold mining area studied has 5 mine tailings that have been accumulated throughout the operating history of the mine. The topography of the area is relatively flat, and the vegetation is largely grassland. Livestock farming is also widespread in the surrounding area. The climate is temperate, with temperatures averaging 24 °C in summer and 13 °C in winter, occasionally dipping below the freezing point. Annual rainfall is about 750 mm [10]. The study area has a worker population of around 14,000. This figure would be much higher if their families were taken into account. There are also some informal settlements residing close to the mine tailings. The control area, geologically similar to the study area, was chosen in the province of North West in South Africa. The study area and the control area are both part of the Witwatersrand Basin, which is a geological formation created mainly from sedimentary rocks. These rocks, known as the “Witwatersrand Supergroup’’ consists of banded ironstones, quartzites, tillites, conglomerates, mudstones, and some marine lava deposits [11].

### 2.2. Sample Collection and Preparation 

A total of 66 soil samples, 56 from 5 mine tailings in the study area and 10 from the control area, were randomly collected with a coring tool at a depth of 5 cm. The sampling points were identified by using Global Positioning System (GPS). The soil samples each measuring 4 kg were then packaged in plastic bags, carrying identification marks according to IAEA [12]. They were then taken to the laboratory for preparation before analysis. At the laboratory, the soil samples were first spread out on a plastic sheet and allowed to air dry for 2–3 days. The soil samples were then heated in an electric oven at 110 °C for up to 24 h to remove moisture content and thereafter put in an electric furnace at 350 °C for 48 h to burn the plant remains [13]. The samples were then passed through a sieve of mesh size 2 mm [14] to obtain a homogenous sample matrix. Close attention was paid to every sample to avoid cross-contamination. Each of the samples was weighed using an electronic balance. The samples, with known masses, were then packed and sealed in plastic marinelli beakers for 28 days in order to establish a secular equilibrium among some progenies of ^238^U and ^232^Th series [15]. 

## 3. Activity Concentrations Measurements

Measurement of activity concentrations in soil samples was carried out by means of a broad energy germanium (BEGe) detector (BE6530) manufactured by Canberra Industries. It has a relative efficiency of 60% and a resolution of 2.0 keV for 1332 keV gamma ray emission of ^60^Co [16]. Energy and efficiency calibrations of the gamma spectrometer were also performed before sample measurement. The gamma photons from the samples were detected by placing the marinelli beaker directly over the BEGe detector, which is usually lead-shielded to avoid background radiation. For the BEGe detector to work, the electronics requires liquid nitrogen, which was used to cool the detector. Each sample was counted for 12 h to allow for measurable activity. The background radiation around the detector inside the shielding was also measured using an empty marinelli beaker under similar measurement geometry. This background was later subtracted from the measured gamma spectrum of each sample before calculating the activity concentrations. The counting geometry was created during efficiency calibration and carefully duplicated for all the measurements. Measuring process and analysis of spectra were computer-controlled using GENIE 2000 software. The activity concentration of ^238^U was determined by measuring the 295.2 keV (19.7%) and 351.9 keV (38.9%) gamma-rays from ^214^Pb and the 609.3 keV (43.3%), 1120.3 keV (15.7%) and 1764.5 keV (15.1%) gamma-rays from ^214^Bi. ^232^Th activity was determined from the gamma rays of 238.6 keV (44.6%) from ^212^Pb and 338.3 keV (11.4%), 911.6 keV (27.7%) and 969.1 keV (16.6%) from ^228^Ac. Potassium-40 concentration was measured from its 1460.8 keV (10.7%) gamma-ray line [12]. Measurement of these samples was done at the Centre for Applied Radiation Science and Technology (CARST).

The activity concentrations of ^238^U, ^232^Th, and ^40^K were determined using Equation (1) [17].

(1) $$A=\frac{N}{\varepsilon_{f}P_{\gamma }t_{s}mK}$$

where:

*N* = the corrected net peak area of the corresponding full-energy peak
N = N_S_−N_B_
*N_S_* = the net peak area in the spectrum of the sample
*N_B_* = the corresponding net peak area in the background spectrum
ε*_f_* = the efficiency at photo peak energy
*t_s_* = the live time of the sample spectrum collection in seconds
*m* = the mass (kg) of the measured sample
*P*_γ_ = the gamma-ray emission probability corresponding to the peak energy
*K* = the correction factor for nuclide decay from the time of sampling to counting

### 3.1. Energy and Efficiency Calibration for Gamma Spectrometry

Energy calibration was accomplished by measuring the spectrum of europium (^152^Eu) point source with known full-peak energies. ^152^Eu was chosen because it consists of multiple energy peaks that cover a large energy region. The ^152^Eu point source with an activity of 5 kBq as of 12:00 noon, 11 June 2008 was placed on the detector and ran for 1 h. This was long enough to identify the peak energies in the spectrum. 

Efficiency calibration was done using Laboratory Sourceless Calibration Software (LabSOCS). This calibration software eliminates the need for standard sources for efficiency calibration [18]. A mathematical template for the detector that was characterized by CANBERRA with a combination of NIST-traceable sources was used. This was a simplified marinelli beaker geometry. After selecting the geometry, all the relevant physical sample parameters required by the template (such as sample fill height, material, density, and distance to the detector) were entered into LabSOCS Calibration Software. An efficiency calibration for the conditions specified was then generated and used for the analysis of the spectrum collected in order to yield qualitative and quantitative results.

### 3.2. Validation of Measurements

In order to make sure that the activity concentrations of the soil samples measured were accurate, the results were compared with activity concentrations of known reference standard materials. The results confirmed that there was secular equilibrium between Radon-222 (^222^Rn) and its progeny. The reference materials used were IAEA standard sources. For ^238^U, ^232^Th and ^40^K, IAEA-RGU-1, IAEA-RGTh-1 and IAEA-RGK-1 reference materials were used, respectively. These reference materials were released on 1 January 1987, and their activity concentrations were confirmed during measurement. 

### 3.3. Absorbed Dose

#### 3.3.1. Radium Equivalent Activity (Ra_eq_)

Radiological hazards in environmental substances are estimated through various hazard parameters. One of the most common radiological indices is called the Radium Equivalent Activity (Ra_eq_), which is the actual activity level of ^238^U, ^232^Th and ^40^K in soil samples [19]. This takes care of the non-uniformity of natural radionuclides in soil samples. It produces the weighted sum of ^238^U (which can be that of ^226^Ra), ^232^Th and ^40^K activity concentrations based on the assumption that 370 Bq∙kg^−1^ of ^226^Ra, 259 Bq∙kg^−1^ of ^232^Th, and 4810 Bq∙kg^−1^ of ^40^K has the same gamma-ray dose rate. The result is that each of these radionuclides produces an effective dose of 1.5 mGy per year [20], which is assumed to be the maximum permissible dose to members of the public from their exposure to natural radiation from soil. On the basis of these values, $Ra_{eq}$ is defined as follows:

(2) $$Ra_{eq}=\left(\frac{A_{Ra}}{370}+\frac{A_{Th}}{259}+\frac{A_{K}}{4810}\right)\times 370$$

which is equivalent to

(3) $$Ra_{eq}=A_{Ra}+1.43A_{Th}+0.077A_{K}$$

where $A_{Ra}$, $A_{Th}$ and $A_{K}$ are the activity concentrations in Bq∙kg^−1^ of ^226^Ra , ^232^Th, and ^40^K, respectively. The $Ra_{eq}$ is therefore a single index or number to describe the gamma output from different mixtures of radionuclides in a material. 

#### 3.3.2. Absorbed Dose Rate in Air (*D*)

For radiation protection purposes, the absorbed dose rate coming from the external gamma radiation can be calculated from the activity concentrations in soil. This outdoor absorbed dose rate in air due to terrestrial gamma rays at 1 m above the ground level is calculated from ^226^Ra (^238^U), ^232^Th and ^40^K concentration values in soil, assuming that the other radionuclides, such as ^137^Cs, ^90^Sr and the ^235^U, are negligible since they contribute very little to the total dose from environmental background [21]. In general, the activity concentrations of ^238^U and ^232^Th are estimated based on the assumption of secular equilibrium conditions between parents and daughter isotopes.

The absorbed dose rate in the air is therefore given by the following formula [22];

(4) $$D(nGy\cdot h^{-1})=0.462A_{Ra}+0.604A_{Th}+0.0417A_{K}$$

where *D* is the absorbed dose rate in air, and $A_{Ra}$, $A_{Th}$ and $A_{K}$ are the activity concentrations of ^226^Ra (^238^U), ^232^Th and ^40^K, respectively. The dose coefficients in units of nGy∙h^−1^ per Bq∙kg^−^^1^ were taken from the UNSCEAR report [22].

#### 3.3.3. Annual Effective Dose Equivalent (*AEDE*)

The absorbed dose rate in air does not provide the radiological risk directly. It is the annual effective dose equivalent from outdoor terrestrial gamma radiation that is used instead. *D* is converted to *AEDE* by making use of a conversion coefficient and the outdoor occupancy factor [20]. Using the conversion coefficient of 0.7 Sv∙Gy^−1^ from the absorbed dose in the air to the effective dose received by adults and an outdoor occupancy factor of 0.2 as proposed by UNSCEAR [22], *AEDE* can be estimated from the following formula:

(5) $$AEDE\left(mSv\cdot y^{-1}\right)=D\left(nGy\cdot h^{-1}\right)\;\times 8760\;h\;\times \;0.2\;\times \;0.7\;Sv\cdot Gy^{-1}\;\times \;10^{-6}$$

where 8760 is the time in hours for one year, and 10^−6^ is the factor converting from nano to milli.

#### 3.3.4. Radiation Hazard Indices

To limit the radiation exposure due to natural radionuclides in the samples to a maximum permissible limit of 1 mSv∙y^−1^, the External Hazard Index (*H_ex_*) was introduced [23]. The value of this *H_ex_* must be less than unity in order to keep the radiation hazard negligible. This means that the maximum value of *H_ex_* must correspond to the upper limit of Radium Equivalent Activity of 370 Bq∙kg^−1^ [24]. This is a widely used hazard index reflecting the external exposure and is defined by Equation (6) as follows [22]:

(6) $$H_{ex}=\left(\frac{A_{Ra}}{370}+\frac{A_{Th}}{259}+\frac{A_{K}}{4810}\right)$$

where $A_{Ra}$, $A_{Th}$ and $A_{K}$ are the mean activities concentration of ^226^Ra (^238^U), ^232^Th and ^40^K, in Bq∙kg^−^^1^, respectively.

In addition to *H_ex_*, radon and its short-lived products may also be hazardous to human beings. Its internal exposure to body organs is quantified by the Internal Hazard Index (*H_in_*), which is given by the Equation (7) [22]:

(7) $$H_{in}=\left(\frac{A_{Ra}}{185}+\frac{A_{Th}}{259}+\frac{A_{K}}{4810}\right)$$

The value of *H_in_* must also be less than unity for the radiation hazard to be negligible.

## 4. Results and Discussion

### 4.1. Activity Concentrations of Natural Radionuclides.

The results presented in Table 1 show average activity concentrations of NORMs analyzed from the gold mine tailings and from the control area. The range of activity concentrations in Bq∙kg^−1^ for ^238^U, ^232^Th and ^40^K from the mine tailings varied from 87.2 ± 1.6 to 2668.9 ± 46.2, 20.5 ± 0.6 to 89.7 ± 1.9 and 226.5 ± 7.8 to 781.0 ± 23.9, respectively. On the other hand, the range of activity concentrations in Bq∙kg^−1^ for ^238^U, ^232^Th, and ^40^K from the control area varied from 12.5 ± 0.3 to 23.6 ± 0.5, 16.6 ± 0.4 to 30.4 ± 0.6 and 424.3 ± 13.0 to 648.4 ± 19.8, respectively. The error in the activity concentrations was calculated using propagation of uncertainty equations based on the weighted average of the radionuclides.

**Table 1:** Activity concentrations of ^238^U, ^232^Th and ^40^K for soil samples from the mine tailings and from the control area.

Location	No. of Samples	Parameter	Activity Concentrations (Bq∙kg^−1^)		
			^238^U	^232^Th	^40^K
Control area	10	Average ± Error	17.0 ± 0.4	22.2 ± 0.5	496.8 ± 15.2
		Min ± Error	12.5 ± 0.3	16.6 ± 0.4	424.3 ± 13.0
		Max ± Error	23.6 ± 0.5	30.4 ± 0.6	648.4 ± 19.8
Tailings one	11	Average ± Error	733.4 ± 12.7	41.3 ± 0.9	339.9 ± 10.7
		Min ± Error	304.4 ± 5.4	36.5 ± 0.8	271.5 ± 9.0
		Max ± Error	1243.4 ± 21.6	54.5 ± 1.2	468.4 ± 14.6
Tailings two	13	Average ± Error	794.9 ± 13.8	44.9 ± 1.0	460.7 ±14.4
		Min ± Error	616.6 ± 10.7	38.4 ± 0.9	226.5 ±7.8
		Max ± Error	1391.8 ± 24.1	49.5 ± 1.1	681.9 ± 21.0
Tailings three	8	Average ± Error	1556.2 ± 27.0	59.0 ± 1.3	354.3 ± 11.4
		Min ± Error	390.9 ± 6.8	22.3 ± 0.6	233.8 ± 7.5
		Max ± Error	2668.9 ± 46.2	89.7 ± 1.9	497.1 ± 15.6
Tailings four	12	Average ± Error	232.0 ± 4.1	33.2 ± 0.8	489.5 ± 14.1
		Min ± Error	87.2 ± 1.6	20.5 ± 0.6	257.9 ± 8.3
		Max ± Error	618.2 ± 10.8	49.1 ± 1.1	781.0 ± 23.9
Tailings five	12	Average ± Error	609.7 ± 10.6	41.1 ± 0.9	490.7 ± 15.2
		Min ± Error	236.1 ± 4.2	25.3 ± 0.6	281.2 ± 8.8
		Max ± Error	2054.7 ± 35.6	67.1 ± 1.4	574.9 ± 17.7
Average all tailings			485.3 ± 13.7	43.9 ± 1.0	427.0 ± 13.1

The results in Table 1 show that the average activity concentration of ^238^U was highest in tailings three, followed by tailings two, tailings one, and then tailings five. The least average activity concentration of ^238^U was found in tailings four. These results, compared to the control area, indicated that mining activities contributed significantly to the elevated levels of ^238^U. For ^232^Th, the results also show enhanced levels of ^232^Th in the gold mine tailings compared to the control area. Mine tailings three had the highest average activity concentration compared to the other tailings mainly because, in other tailings, gold ore had been reprocessed to extract uranium. The average activity concentration of ^40^K in all the mine tailings was comparable to that from the control area. ^40^K activity concentration from the control area would have been expected to be lower than that from mine tailings. However, this was not the case, and it is believed that the control area could have been used for agricultural production many years back. The use of fertilizers in agriculture may be responsible for this increase.

The activity concentrations of ^238^U, ^232^Th and ^40^K in soils from the mine tailings were also compared with results published from other countries and the results are presented in Table 2. The average activity concentration of ^238^U from the study area was very high compared to the other countries (including uranium areas) and the world average. This is attributed to the large volumes of uranium waste found in the gold mine, estimated to be almost ten times the amount of gold itself [7]. For ^232^Th and ^40^K, the average activity concentration from the mine tailings was comparable to the world average.

**Table 2:** Comparison of activity concentrations of ^238^U, ^232^Th and ^40^K in soils from the study area with other countries.

Country	Activity Concentrations in Soil (Bq∙kg^−1^)					
	^238^U		^232^Th		^40^K	
	Mean	Range	Mean	Range	Mean	Range
South Africa (**)	485.3	87.2–2668.9	43.9	20.5–89.7	427.0	226.5–781.0
Nigeria (^**++**^)	37	6–67	63	17–87	998	85–1565
Ghana (*)	29	-	25	-	582	-
Egypt (^**+**^)	37	6–120	18	2–96	320	29–650
Algeria (^**+**^)	30	2–110	25	2–140	370	66–1150
United States (^**+**^)	35	4–140	35	4–130	370	100–700
Romania (Uranium area) (^**+**^)	57	-	31	-	486	-
China (^**+**^)	33	2–690	41	1–360	440	9–1800
India (^**+**^)	29	7–81	64	14–160	400	38–760
Japan (^**+**^)	29	259	28	2–288	310	15–990
Syrian Arab Republic (^**+**^)	23	10–64	20	10–32	270	87–780
Ireland (^**+**^)	37	8–120	26	3–60	350	40–800
Switzerland (^**+**^)	40	10–150	25	4–70	370	40–1000
Hungary (^**+**^)	29	12–66	28	12–45	370	79–570
Russian Federation (^**+**^)	19	0–67	30	2–79	520	100–1400
Greece (^**+**^)	25	1–240	21	1–190	360	12–570
Portugal (^**+**^)	49	26–82	51	22–100	840	220–1230
World average (^**+**^)	33		45		420	

Notes: (**^+^**) [22], (**^++^**) [15]; (*) [25]; (**) Present work.

### 4.2. Radiological Hazard Assessment

All the radiological hazard parameters were considered for the study area and the results of the findings are summarized in Table 3.

**Table 3:** Calculated Radium Equivalent Activity (*Ra_eq_*), Absorbed Dose Rate in air (*D*), Annual Effective Dose Equivalent (*AEDE*), External Hazard Index (*H_ex_*) and Internal hazard Index (*H_in_*) of soil samples from the mine tailings and from the control area.

Location	No. of Samples	Parameter	*Ra_eq_* (Bq∙kg^−1^)	*D* (nGy∙h^−1^)	*AEDE* (mSv∙y^−1^)	*H_ex_*	*H_in_*
Control area	10	Average	86.9 ± 1.4	41.9 ± 0.7	0.1	0.2	0.3
		Min	76.4 ± 1.4	37.5 ± 0.7	0.0	0.2	0.2
		Max	103.8 ± 1.5	49.1 ± 0.8	0.1	0.3	0.3
Tailings one	11	Average	818.6 ± 12.9	377.9 ± 5.9	0.5	2.2	4.2
		Min	390.8 ± 5.6	181.1 ± 2.6	0.2	1.1	1.9
		Max	1342.2 ± 21.7	618.7 ± 10.0	0.8	3.6	7.0
Tailings two	13	Average	894.6 ± 14.0	413.6 ± 6.4	0.5	2.4	4.6
		Min	707.2 ± 10.9	327.3 ± 5.0	0.4	1.9	3.6
		Max	1480.0 ± 24.2	682.3 ± 11.2	0.8	4.0	7.8
Tailings three	8	Average	1667.9 ± 27.1	769.4 ± 12.5	0.9	4.5	8.7
		Min	440.9 ± 6.9	203.9 ± 3.2	0.3	1.2	2.2
		Max	2811.9 ± 46.3	1297.3 ± 21.4	1.6	7.6	14.8
Tailings four	12	Average	317.1 ± 4.4	147.6 ± 2.1	0.2	0.9	1.5
		Min	160.3 ± 2.1	74.5 ± 1.0	0.1	0.4	0.7
		Max	746.7 ± 11.0	346.9 ± 5.1	0.4	2.0	3.7
Tailings five	12	Average	706.4 ± 10.8	327.0 ± 5.0	0.4	1.9	3.6
		Min	317.8 ± 4.4	148.0 ± 2.1	0.2	0.9	1.5
		Max	2182.1 ± 35.7	1007.3 ± 16.5	1.2	5.9	11.5
Average all tailings			880.9 ± 13.8	407.1 ± 6.4	0.5	2.4	4.5
Worldwide average			370	59	0.48	<1	<1

#### 4.2.1. Radium Equivalent Activity (*Ra_eq_*)

The Ra_eq_ average values for the mine tailings and the control area ranged from 160.3 ± 2.1 to 2811.9 ± 46.3 Bq∙kg^−^^1^ with an average of 880.9 ± 13.8 Bq∙kg^−^^1^ and from 76.4 ± 1.4 to 103.8 ± 1.5 Bq∙kg^−^^1^ with an average of 86.9 ± 1.4 Bq∙kg^−^^1^ respectively. The minimum and the maximum values were as a result of tailings four and tailings three respectively. Tailings four is operational, and both gold and uranium is being extracted, while tailings three is a disused storage facility that has not been reprocessed for uranium. In most of the soil samples from the mine tailings, *Ra_eq_* values were above the worldwide value of 370 Bq∙kg^−^^1^ recommended under normal circumstances [26], rendering the area around the mine tailings unsafe. This has been attributed by the high levels of ^238^U found in the mine tailings.

#### 4.2.2. Absorbed Dose Rate in Air (D)

From the calculations in Table 3, the outdoor average dose rate in air from terrestrial gamma rays was found to be 407.1 ± 6.4 nGy·h^−1^. This is much higher than the worldwide average of 59 nGy∙h^−1^ [22]. However, the value from the control area was found to be 41.9 ± 0.7 nGy∙h^−1^ and is lower than the worldwide average.

#### 4.2.3. Annual Effective Dose Equivalent (AEDE)

The AEDE values for the mine tailings and the control area were also calculated as shown in Table 3. They were found to be in the range 0.1 to 1.6 mSv∙y^−1^ with an average of 0.5 mSv∙y^−1^ and from 0 to 0.1 mSv∙y^−1^ with an average of 0.1 mSv∙y^−1^ for the mine tailings and the control area, respectively. Although some AEDE values were below the worldwide average of 0.48 mSv [22], it is also important to note that there were also some samples above the worldwide average. The International Commission on Radiation Protection (ICRP) recommends the AEDE limit of 1 mSv∙y^−1^ for individual members of the public and 20 mSv∙y^−1^ for radiation workers [23]. In South Africa, the dose constraint applicable to the average member of a critical group from a single source within the exposed population is 0.25 mSv per annum [27]. This means that the *AEDE* average values from the mine tailings were considered unsafe to the population, but those from the control area were below the regulatory limit. 

#### 4.2.4. Radiation Hazard Indices

Calculated values of hazard indices for soil samples from mine tailings ranged from 0.4 to 7.6 with an average of 2.4 and from 0.7 to 14.8 with an average of 4.5 for the external hazard (*H_ex_*) and internal hazard (*H_in_*), respectively. This shows that the average values for *H_ex_* and *H_in_* were higher than unity, posing a significant radiological threat to the population in the area.

## 5. Conclusions 

The natural radioactivity levels of ^238^U, ^232^Th and ^40^K have been measured in 56 soil samples from the gold mine tailings and 10 soil samples from the control area using gamma spectrometry using broad energy germanium detector (BE6530). The average activity concentrations in Bq∙kg^−1^ for ^238^U from the mine tailings were comparably higher than the worldwide average. However, those of ^232^Th and ^40^K were comparable to the worldwide average and other countries. The average values for Raeq from the mine tailings were also calculated and observed to be higher than 370 Bq∙kg^−1^, which is a recommended safe limit to avoid exposure to radiation hazards from the area. The outdoor average dose rate in air from terrestrial gamma rays was also found to be higher than the worldwide average of 59 nGy∙h^−1^ [22]. Calculated average *AEDE* values from the mine tailings for members of the public were also above the world average. The radiological hazard indices calculated in the present work were higher than unity, presenting a potential radiological hazard to the population in the area. The average values for the external hazard (*H_ex_*) and internal hazard (*H_in_*) from the mine tailings were found to be 2.4 and 4.5 respectively. The results obtained in this study have established baseline information on natural radioactivity, which can be used as a reference point for future work in the area.
